# Exploring the therapeutic potential of *Asparagus africanus* in polycystic ovarian syndrome: a computational analysis

**DOI:** 10.1515/jib-2024-0019

**Published:** 2024-12-12

**Authors:** Sania Riaz, Fatima Haider, Rizwan- ur-Rehman, Aqsa Zafar

**Affiliations:** Department of Bioinformatics and Biosciences, Capital University of Science and Technology, Islamabad, Pakistan

**Keywords:** *Asparagus africanus*, follicle-stimulating hormone receptor, luteinizing hormone receptor, molecular docking, polycystic ovarian syndrome

## Abstract

PCOS is a multifaceted condition characterized by ovarian abnormalities, metabolic disorders, anovulation, and hormonal imbalances. In response to the growing demand for treatments with fewer side effects, the exploration of herbal-origin drugs has gained prominence. *Asparagus africanus*, a traditional medicinal plant that exhibits anti-inflammatory, antioxidant, and anti-androgenic properties may have a cure for PCOS. The plant has rich biochemical profile prompted its exploration as a potential source for drug development. The aim of this study is to investigate the potential therapeutic efficacy of *A. africanus* in the management of PCOS through molecular docking studies with Luteinizing Hormone Receptor and Follicle-Stimulating Hormone Receptor proteins. The identified compounds underwent molecular docking against key proteins associated with PCOS, namely Luteinizing Hormone Receptor and Follicle-Stimulating Hormone Receptor. The results underscored the lead compound’s superiority, demonstrating favorable pharmacokinetics, ADME characteristics, and strong molecular binding without any observed toxicity in comparison to standard drug. This study, by leveraging natural compounds sourced from *A. africanus*, provides valuable insights and advances towards developing more effective and safer treatments for PCOS. The findings contribute to the evolving landscape of PCOS therapeutics, emphasizing the potential of herbal-origin drugs in mitigating the complexities of this syndrome.

## 1 Introduction

Polycystic Ovarian Syndrome (PCOS) is a widely widespread reproductive condition that affects women within their reproductive years. PCOS is characterized by the presence of numerous follicles (≥12 follicles) with a diameter of less than 9 mm or a volume exceeding 10 ml as determined using ultrasonography [1]. Despite the fact that numerous researches related to epidemiology have investigated the prevalence of PCOS, their findings vary due to the difference of race and ethnicity, study populations, and phenotypes [2]. According to National Institutes of Health (NIH), it is estimated that 4–10 % of women of reproductive age are expected to develop PCOS globally [3].

The development of PCOS involves the dysregulation of luteinizing hormone (LH) and follicle-stimulating hormone (FSH) from the pituitary gland. In individuals with PCOS, there is a modification in the LH/FSH ratio. Elevated LH secretion relative to FSH can lead to heightened androgen production by theca cells, resulting in anovulatory cycles. The surplus of androgens can alter the regulation of female hormones, leading to elevated estrogen levels, menstrual irregularities, and infertility. This hormonal imbalance in the pathogenesis of PCOS plays a significant role [4].

In PCOS, various hormonal imbalances have been identified. Hormones such as insulin, growth hormone, ghrelin, LEAP-2, gonadotropin-releasing hormone (GnRH), the luteinizing hormone/follicle-stimulating hormone (LH/FSH) ratio, androgens, and estrogens are all found to be abnormal in women with PCOS. These hormonal disruptions are associated with metabolic issues like diabetes, insulin resistance, obesity, infertility, and irregular menstrual cycles. Specifically, increased insulin levels, reduced GH, elevated ghrelin, and leptin resistance contribute to a higher risk of diabetes and obesity in PCOS patients. Additionally, decreased GH, elevated LEAP-2 levels, a higher LH/FSH ratio, elevated androgens, and low estrogen levels are all observed in PCOS and are linked to infertility in affected women [5].

Excessive LH levels promote the production of ovarian androgens, while a relative lack of FSH hampers follicular development. This imbalance in the LH:FSH ratio results in the proliferation of ovarian theca cells, enhancing steroid production and leading to hyperandrogenism in women with PCOS. Several genetic factors have been linked to abnormal steroidogenesis, with CYP genes playing a crucial role in androgen synthesis, making them key contributors to the development of hyperandrogenism in PCOS [6].

Autophagy has a complex role in regulating ovarian follicles, affecting both their development and degeneration while contributing to reproductive health by interacting with key hormones like progesterone and estrogen. In PCOS, autophagy becomes dysregulated, leading to disrupted folliculogenesis, reduced granulosa cell populations, and metabolic imbalances. The regulation of autophagy in PCOS is cell-specific, with increased activity in granulosa cells and reduced activity in theca cells, which contributes to varying effects on cell survival and disease progression. This highlights the intricate nature of autophagy’s involvement in PCOS. Additionally, disruptions in circadian rhythms are linked to insulin resistance and hyperandrogenism, key factors in PCOS development. Research shows that circadian rhythms influence autophagy by regulating important autophagy genes and proteins, such as BECN1 and LC3, which are controlled by circadian cycles. Moreover, proteins like SIRT1 have been found to regulate both autophagy and circadian rhythms, affecting metabolic and reproductive functions in PCOS. Despite this, the exact mechanisms by which circadian rhythms affect autophagy in the ovarian tissue during PCOS remain largely unexplored. Understanding this interaction could reveal new pathways in PCOS development, but more research is needed to fully uncover the link between autophagy and circadian biology in the disease [7].

Currently used standard drugs against PCOS have certain side effects. So to avoid these side effects herbal chemicals or phytoestrogens are best to treat PCOS [4]. *Asparagus africanus* is one of the numerous plants in Ethiopia known for its medicinal properties. Belonging to the Family *Liliaceae*, this perennial climbing or erect shrub thrives at altitudes ranging from 700 to 3,800 m above sea level. While it is widely distributed, it particularly flourishes at higher altitudes, reaching up to 6 m within the altitude range of 1,450–2,900 m. Commonly known as “Saritti,” this plant has a longstanding reputation for serving as a traditional remedy for reproductive health issues [8].

The roots of *A. africanus* are used in an infusion as a remedy for venereal diseases. Additionally, during childbirth, women in certain regions, particularly in the rural areas of Bale zone in southern Ethiopia, consume an infusion of the root mixed with water to facilitate the birthing process. Notably, in some traditional practices, the leaves or roots of *Asparagus* spp. are chewed to aid in childbirth [8].

*Asparagus racemosus*, a member of the *Asparagaceae* family, holds a traditional place in Indian medicine, particularly in Ayurveda. Its usage is associated with promoting the normal development of ovarian follicles, regulating the menstrual cycle, and revitalizing the female reproductive system. The beneficial effects are attributed, in part, to its phytoestrogen content, representing natural plant-based estrogens. Additionally, *A. racemosus* is noted for its role in combating hyperinsulinemia [9]. Thus, *A. racemosus* has beneficial effects against PCOS, it is possible that *A. africanus* has them as well, as they both are from related family.

*A. africanus,* a potential management strategy for PCOS identified through computational analysis, warrants further investigation in clinical settings to validate its effectiveness, contributing to holistic and effective treatment strategies for PCOS management.

## 2 Materials and methods

The methodological framework for evaluating *A. africanus* as a potential remedy for PCOS encompasses computational approach.

### 2.1 Disease selection

PCOS is the most prevalent endocrinological condition, impacting women in their reproductive age globally. The condition can be identified by elevated levels of insulin, excessive production of male hormones, irregular menstrual cycles, and persistent metabolic imbalances in females [10, 11]. Several synthetic medications, including metformin, spironolactone, and clomiphene, are utilized for the treatment of PCOS. However, these synthetic drugs are associated with adverse effects such as congenital heart disease [12]. Hence, it is imperative to acquire further understanding regarding the disease.

### 2.2 Selection of target proteins

The majority of women with PCOS exhibit an abnormal ratio of LH and FSH, which is caused by elevated baseline levels and heightened frequency of LH pulses [13, 14]. The LH receptor (LHR), primarily situated on theca cells in the ovary or Leydig cells in the testes, and the FSH receptor (FSHR), found on granulosa cells in the ovary or Sertoli cells in the testes, serve as pivotal translators of FSH and LH signals into endocrine effects. LHR plays a key role in steroid hormone biosynthesis, while FSHR governs the recruitment and maturation of gonadal stem cells, along with the processing and transport of steroid hormones [15, 16]. Consequently, LHR and FSHR have been strategically chosen as the target proteins for this study.

### 2.3 Primary sequence retrieval

Primary sequence of target proteins (LHR and FSHR) are retrieved in FASTA format from UniProt (https://www.uniprot.org/) under accession number P22888 and P23945. These sequences have lengths of 699 and 695 residues, respectively.

### 2.4 Analysis of physicochemical properties

The physiochemical properties of proteins are crucial factors in defining their functional roles. Thus, the physicochemical parameters for LHR and FSHR are determined using the ProtParam tool (https://web.expasy.org/protparam/). The tool computes molecular weight, the number of positively charged residues (Arg + Lys) and negatively charged residues (Asp + Glu), theoretical isoelectric point (pI), extinction coefficient (including Cys), extinction coefficient (without Cys), instability index, aliphatic index, and grand average of hydropathicity.

### 2.5 Retrieval of protein structure

3D structure of LHR and FSHR are retrieved from Protein Data Bank (PDB) (https://www.rcsb.org/). The PDB entry ID for LHR is 7FII, with a resolution of 4.30 Å and entry ID for FSHR is 8I2H, with a resolution of 6.00 Å.

### 2.6 Retrieval of ligand and standard drug structure

The ligands that constitute in *A. africanus* and FDA approved standard drug 3D structures are obtained from PubChem (https://pubchem.ncbi.nlm.nih.gov/) and ChemSpider (http://www.chemspider.com/). The 9 selected phytochemicals in *A. africanus* are 2(3H)-furanose,dihydro-3-hydroxy-4,4-dimethyl (FDHD), Tetramethylpyrazine (TMP), 1,2-Benzenedicarboxylic acid (Phthalic acid), 7,9-Die-tert-butyl-1-oxaspiro(4,5) deca-6,9-diene-2,8-dione (DTBO), n-Hexadecanoic acid (Palmitic acid), Butyl citrate, 3-Dehydro-des-N-26-methyl-dihydro-pseudotomatidine (DMDP), Stigmasterol and Sarsasapogenin [17]. Whereas, the standard drug used commonly for the management of PCOS selected is Clomiphene citrate [18].

### 2.7 Ligand ADME properties and toxicity

The selected ligands are screened through Swiss ADME (http://www.swissadme.ch/) to calculate physicochemical descriptors and provide predictions for ADME parameters. Furthermore, toxicity for selected ligands is predicted trough Protox-2 (https://tox-new.charite.de/).

### 2.8 Active site determination and molecular docking

The target proteins active site will be determined through of Castp (http://sts.bioe.uic.edu/). Further, the ligands and drug will be docked with the target proteins using CB-Dock (http://cao.labshare.cn/cb-dock/) and protein interactions are visualized through BIOVIA Discovery Studio Visualizer [19].

### 2.9 Lead compound identification

The most active inhibitor was determined after conducting a thorough investigation of protein and ligand interactions, ADME properties, and toxicological evaluations.

## 3 Results

An *insilico* evaluation performed to explore the potential therapeutic effects of phytoconstituents derived from *A. africanus* against PCOS.

### 3.1 Protein retrieval

#### 3.1.1 Primary sequence

The initial step involved the acquisition of the primary sequences of the target proteins, LHR and FSHR procured in FASTA format. This retrieval was conducted through UniProt database at (http://www.uniprot.org), utilizing the UniProt accession numbers P22888 and P23945 for LHR and FSHR, respectively. Notably, the primary sequence data indicated that LHR is comprised of 699 residues, while FSHR consists of 695 residues. The lengths of these proteins bear significance due to their potential influence on various functional aspects.

#### 3.1.2 Physicochemical properties of proteins

To assess the physiochemical characteristics of LHR and FSHR, the ProtParam online tool (https://web.expasy.org/protparam/) was employed.

The findings of the physiochemical analysis (Table 1), revealed notable characteristics of the target proteins LHR and FSHR. The molecular weights of these proteins are determined to be 78,642.97 and 78,265.01, respectively. Moving to the isoelectric point (pI), LHR demonstrates a value of 8.82, indicative of its basic nature, while FSHR possesses a pI of 6.77, suggesting an acidic nature. A comparative analysis of charged residues reveals that LHR has a slightly lower count of negatively charged residues compared to FSHR. Conversely, in the realm of positively charged amino acids, LHR exhibits a higher quantity than FSHR. The extinction coefficients, assessed at a wavelength of 280 nm in water, provide insights into the proteins’ absorbance properties. For LHR, extinction coefficient 1 yields a value of 67,740 M^−1^ cm^−1^, while extinction coefficient 2 is calculated at 66,240 M^−1^ cm^−1^. In the case of FSHR, the corresponding values are 68,645 M^−1^ cm^−1^ and 67,270 M^−1^ cm^−1^, respectively.

**Table 1:** Physiochemical properties of LHR and FSHR.

Physicochemical properties	LHR	FSHR
Molecular weight	78,642.97	78,265.01
Theoretical pI	8.82	6.77
Negatively charged residues (Asp + Glu)	53	59
Positively charged residues (Arg + Lys)	68	56
Ext. coefficient 1	67,740	68,645
Ext. coefficient 2	66,240	67,270
Instability index	39.74	43.41
Aliphatic index	100.06	108.73
Gravy	0.171	0.227

Moving on to stability indices, LHR and FSHR present values of 39.74 and 43.41, respectively. Additionally, the aliphatic index, a measure of protein thermostability, is determined to be 100.06 for LHR and 108.73 for FSHR. Lastly, the GRAVY score, indicative of hydrophobicity, is calculated to be 0.171 for LHR and 0.227 for FSHR. These comprehensive physiochemical insights contribute significant understanding of the structural and functional properties of LHR and FSHR, offering valuable information for further investigations.

#### 3.1.3 Protein 3D structure retrieval

**Figure 1:**
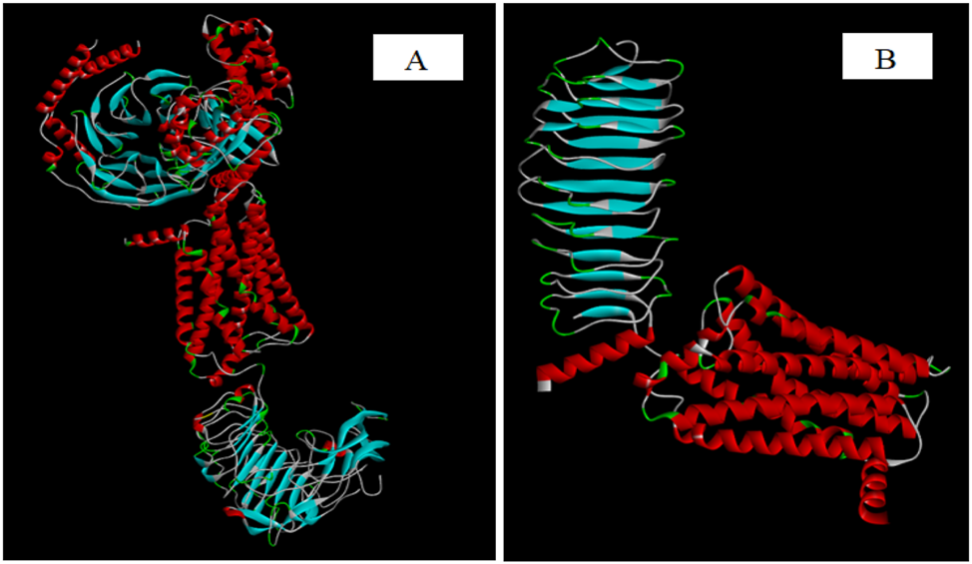
3D structure of proteins. (A) Protein structure representing LHR and (B) protein structure representing FSHR.

In this study, the target proteins, LHR and FSHR, were sourced from the PDB (https://www.rcsb.org/) with entry IDs 7FII and 8I2H (Figure 1). The choice of PDB as the data source ensures the reliability and accuracy of the structural information. Notably, the advantage of utilizing these PDB entries is that the retrieved proteins, LHR and FSHR, do not necessitate additional purification steps. They are inherently devoid of undesirable molecules such as water molecules and ligands.

### 3.2 Ligand retrieval

#### 3.2.1 Ligand chemical structure

A review of the literature led to the identification of 9 phytoconstituents associated with *A. africanus* [17]. The extraction of ligand 3D structures from *A. africanus* is executed through the utilization of two prominent databases, PubChem (https://pubchem.ncbi.nlm.nih.gov/) and ChemSpider (http://www.chemspider.com/) (Table 2).

**Table 2:** Ligand structures.

Database	Ligand name/ID	Molecular formula	Molecular weight	Ligand structure
PubChem	FDHD/439368	C_6_H_10_O_3_	130.14 g/mol	**Figure:** 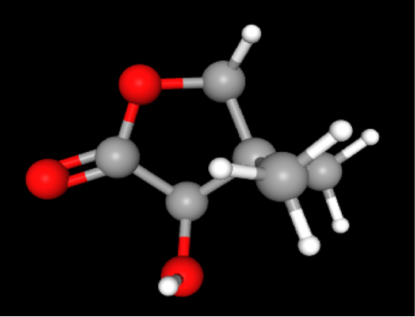
	TMP/14296	C_8_H_12_N_2_	136.19 g/mol	**Figure:** 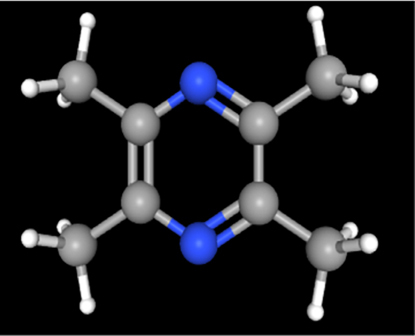
	Phthalic acid/1017	C_16_H_22_O_4_	278.34 g/mol	**Figure:** 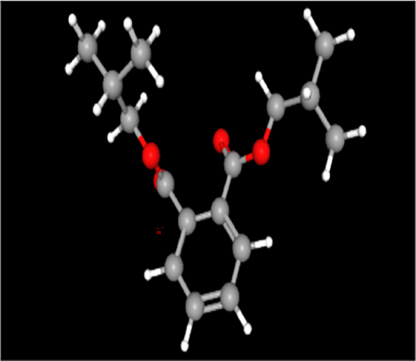
	DTBO/545303	C_17_H_24_O_3_	276.4 g/mol	**Figure:** 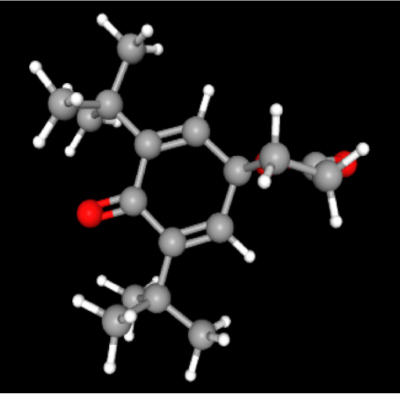
	Palmitic acid/985	C_16_H_32_O_2_	256.42 g/mol	**Figure:** 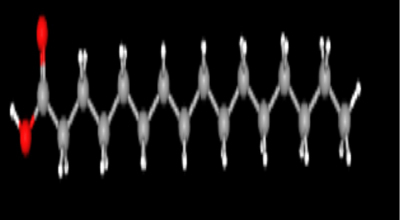
	Stigmasterol/5280794	C_29_H_48_O	412.7 g/mol	**Figure:** 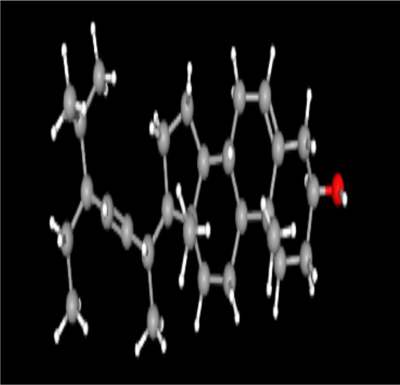
	Sarsasapogenin/92095	C_27_H_44_O_3_	416.6 g/mol	**Figure:** 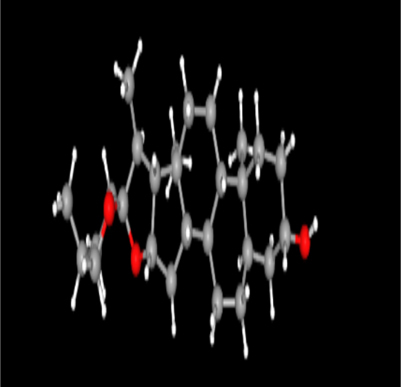
ChemSpider	Butyl citrate/6261	C_18_H_32_O_7_	360.443 Da	**Figure:** 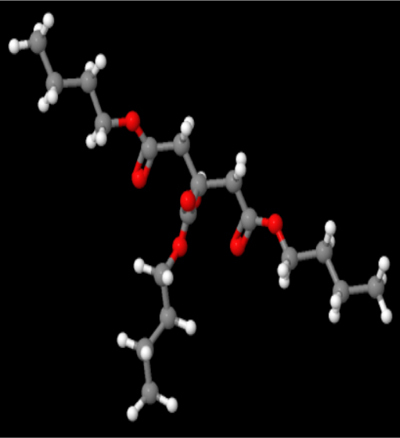
	DMDP/510157	C_28_H_46_O_2_	414.664 Da	**Figure:** 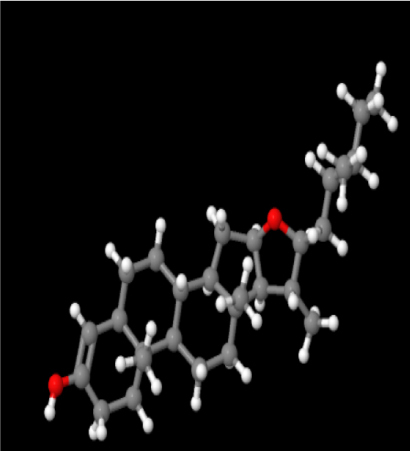

#### 3.2.2 ADME properties

The SwissADME is an internet-based tool that computes significant pharmacokinetic, physicochemical, drug-like, and related parameters for one or many compounds. This is a valuable bioinformatics expertise for the initial drug development stages [20]. SwissADME (http://www.swissadme.ch/) is employed to evaluate ligands in order to identify the most suitable molecule for drug development.

**Figure 2:**
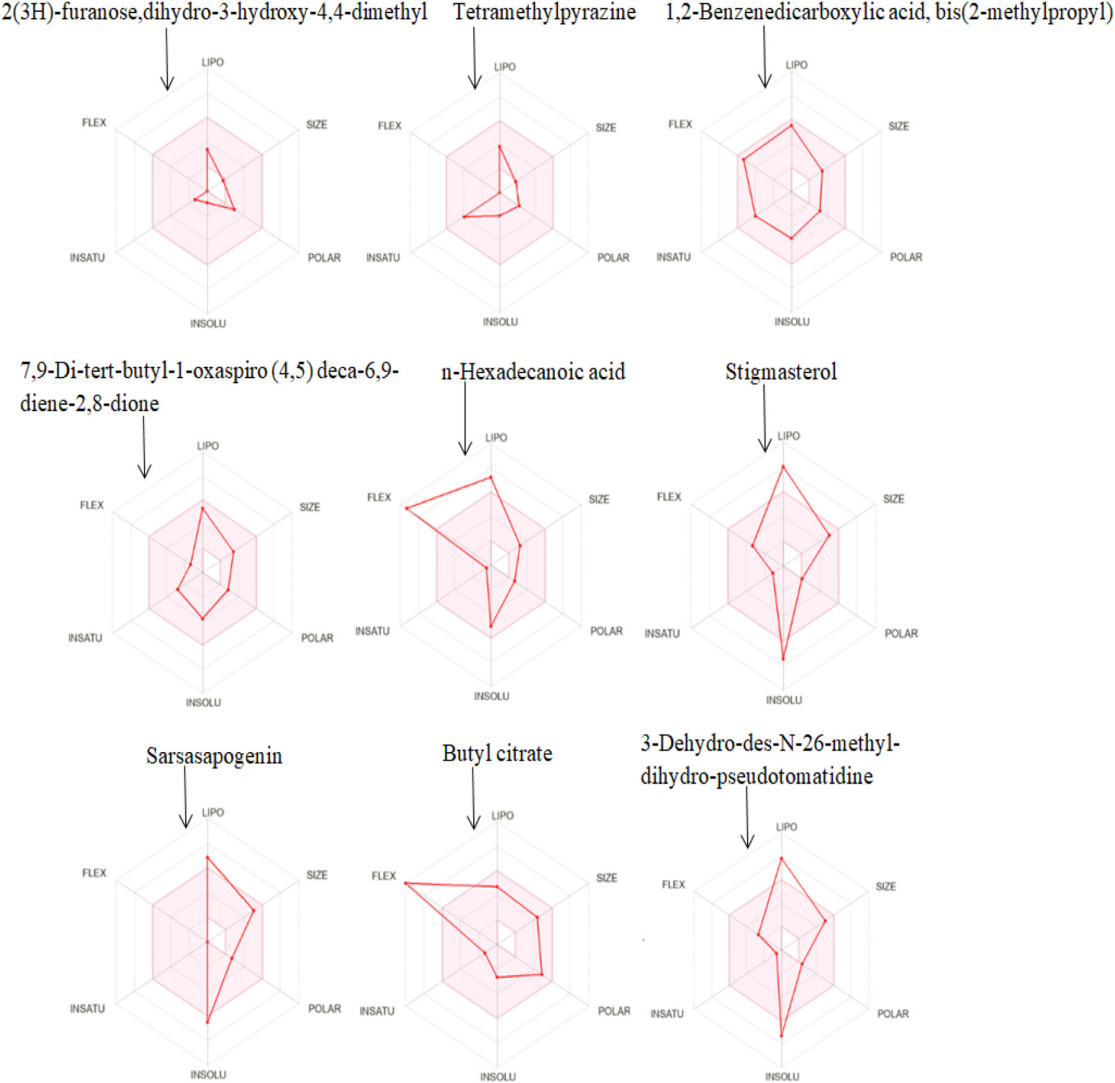
Bioavailability radar of ligands.

The bioavailability radar gives an early assessment of the drug-likeness of the compounds of interest (Figure 2). The pink region serves as an illustration of the optimal physicochemical space for all predicted orally bioavailable properties. Polarity (POLAR), Lipophilicity (LIPO), Insolubility (INSOLU), Flexibility (FLEX) and Insaturation (INSATU) are the six physicochemical properties that are considered [21].

Physicochemical characteristics (Table 3) include the molecular weight, molecular formula, number of heavy atoms, fraction csp3, number of aromatic heavy atoms, number of rotatable bonds, number of H-bond donors, number of H-bond acceptors, molar refractivity, and Topological Polar Surface Area (TPSA) [21].

**Table 3:** Physicochemical properties of ligands.

Ligands	Physicochemical properties									
	Formula	MW (g/mol)	No. of HA	No. of AHA	Fraction Csp3	No. RB	No. of HBA	No. of HBD	MR	TPSA
FDHD	C_6_H_10_O_3_	130.14	9	0	0.83	0	3	1	31.03	46.53 Å^2^
TMP	C_8_H_12_N_2_	136.19	10	6	0.50	0	2	0	41.90	25.78 Å^2^
Phthalic acid	C_16_H_22_O_4_	278.34	20	6	0.50	8	4	0	77.84	52.60 Å^2^
DTBO	C_17_H_24_O_3_	276.37	20	0	0.65	2	3	0	79.66	43.37 Å^2^
Palmitic acid	C_16_H_32_O_2_	256.42	18	0	0.94	14	2	1	80.80	37.30 Å^2^
Stigmasterol	C_29_H_48_O	412.69	30	0	0.86	5	1	1	132.75	20.23 Å^2^
Sarsasapogenin	C_27_H_44_O_3_	416.64	30	0	1.00	0	3	1	122.07	38.69 Å^2^
Butyl citrate	C_18_H_32_O_7_	360.44	25	0	0.83	17	7	1	93.69	99.13 Å^2^
DMDP	C_28_H_46_O_2_	414.66	30	0	0.93	4	2	1	127.80	29.46 Å^2^

MW: molecular weight, No. of HA: number of heavy atoms, No. of AHA: number of aromatic heavy atoms, No. RB: number of rotatable bonds, No. of HBA: number of hydrogen bond acceptor, No. of HBD: number of hydrogen bond donor, MR: molar refractivity, TPSA: topological polar surface area.

Lipophilicity is an important element in drug discovery and design since it is the most informative and effective physicochemical parameter in medicinal chemistry (Table 4). SwissADME offers five publicly accessible models, namely WLOGP, XLOGP3, SILICOS-IT, MLOGP and iLOGP, to assess the lipophilicity of a compound. XLOGP3 is an atomistic approach that incorporates corrective factors and a knowledge-based library. WLOGP is an atomistic method that focuses on fragmental systems. MLOGP is a topological method that relies on a linear relationship with 13 implemented molecular descriptors. SILICOS-IT is a hybrid approach that uses 27 pieces and 7 topological descriptors. iLOGP is a physics-based approach that calculates the free energy of solvation in n-octanol and water using the generalized-born and solvent accessible surface area (GB/SA) model. The consensus log Po/w is calculated by taking the arithmetic average of the values predicted by the five proposed methodologies [21].

**Table 4:** Lipophilicity properties of ligands.

Ligands	Lipophilicity					
	Log Po/w (iLOGP)	Log Po/w (XLOGP3)	Log Po/w (WLOGP)	Log Po/w (MLOGP)	Log Po/w (SILICOS-IT)	Consensus Log Po/w
FDHD	1.13	0.46	−0.07	0.00	0.78	0.46
TMP	1.99	1.28	1.71	0.55	2.66	1.64
Phthalic acid	3.31	4.11	3.31	3.43	3.63	3.56
DTBO	2.91	3.81	3.59	2.87	3.82	3.4
Palmitic acid	3.85	7.17	5.55	4.19	5.25	5.2
Stigmasterol	5.01	8.56	7.8	6.62	6.86	6.97
Sarsasapogenin	4.42	6.49	5.79	5.08	4.3	5.22
Butyl citrate	3.84	2.72	2.53	1.92	3.75	2.95
DMDP	4.57	8.21	7.54	5.61	5.57	6.3

The water solubility (Table 5) prediction methods employed by SwissADME include the ESOL model [22], Ali [23], and SILICOS-IT. ESOL [22] is a model used to determine the solubility of a substance. The solubility is measured on a logarithmic scale, with values ranging from insoluble (<−10) to poorly soluble (<−6), moderately soluble (<−4), soluble (<−2), and highly soluble (0 or higher). Ali [23] solubility class is Log S, which indicates the scale of solubility. The scale ranges from insoluble (<−10) to poorly soluble (<−6), moderately soluble (<−4), soluble (<2), and highly soluble (<0). SILICOS-IT is classified based on solubility using the Log S Scale. It is categorised as insoluble when the Log S value is below −10, weakly soluble when it is between −10 and −6, moderately soluble when it is between −6 and −4, soluble when it is between −4 and −2, and highly soluble when it is between −2 and 0. SwissADME also offers measurements of solubility in mol L^−1^ and mg mL^−1^, as well as qualitative classes of solubility [21].

**Table 5:** Water solubility of ligands.

Ligands	Water solubility											
	Log S (ESOL)	Solubility (ESOL)		Class (ESOL)	Log S (Ali)	Solubility (Ali)		Class (Ali)	Log S (Silicos-IT)	Solubility (Silicos-IT)		Class (Silicos-IT)
		mg mL^−1^	mol L^−1^			mg mL^−1^	mol L^−1^			mg mL^−1^	mol L^−1^	
FDHD	−0.94	1.51E+01	1.16E−01	VS	−1.01	1.28E+01	9.87E−02	VS	−0.54	3.76E+01	2.89E−01	S
TMP	−1.93	1.58E+00	1.16E−02	VS	−1.42	5.17E+00	3.79E−02	VS	−3.17	9.21E−02	6.76E−04	S
Phthalic acid	−3.85	3.94E−02	1.42E−04	S	−4.92	3.34E−03	1.20E−05	MS	−4.23	1.64E−02	5.90E−05	MS
DTBO	−3.82	4.17E−02	1.51E−04	S	−4.42	1.06E−02	3.84E−05	MS	−3.81	4.33E−02	1.57E−04	S
Palmitic acid	−5.02	2.43E−03	9.49E−06	MS	−7.77	4.31E−06	1.68E−08	PS	−5.31	1.25E−03	4.88E−06	MS
Stigmasterol	−7.46	1.43E−05	3.46E−08	PS	−8.86	5.71E−07	1.38E−09	PS	−5.47	1.40E−03	3.39E−06	MS
Sarsasapogenin	−6.51	1.28E−04	3.08E−07	PS	−7.1	3.32E−05	7.97E−08	PS	−4.51	1.29E−02	3.10E−05	MS
Butyl citrate	−2.67	7.77E−01	2.16E−03	S	−4.46	1.26E−02	3.50E−05	MS	−3.99	3.70E−02	1.03E−04	S
DMDP	−7.32	1.99E−05	4.79E−08	PS	−8.69	8.48E−07	2.04E−09	PS	−5.3	2.06E−03	4.98E−06	MS

VS: very soluble, S: soluble, MS: moderate solubility, PS: poor solubility.

**Figure 3:**
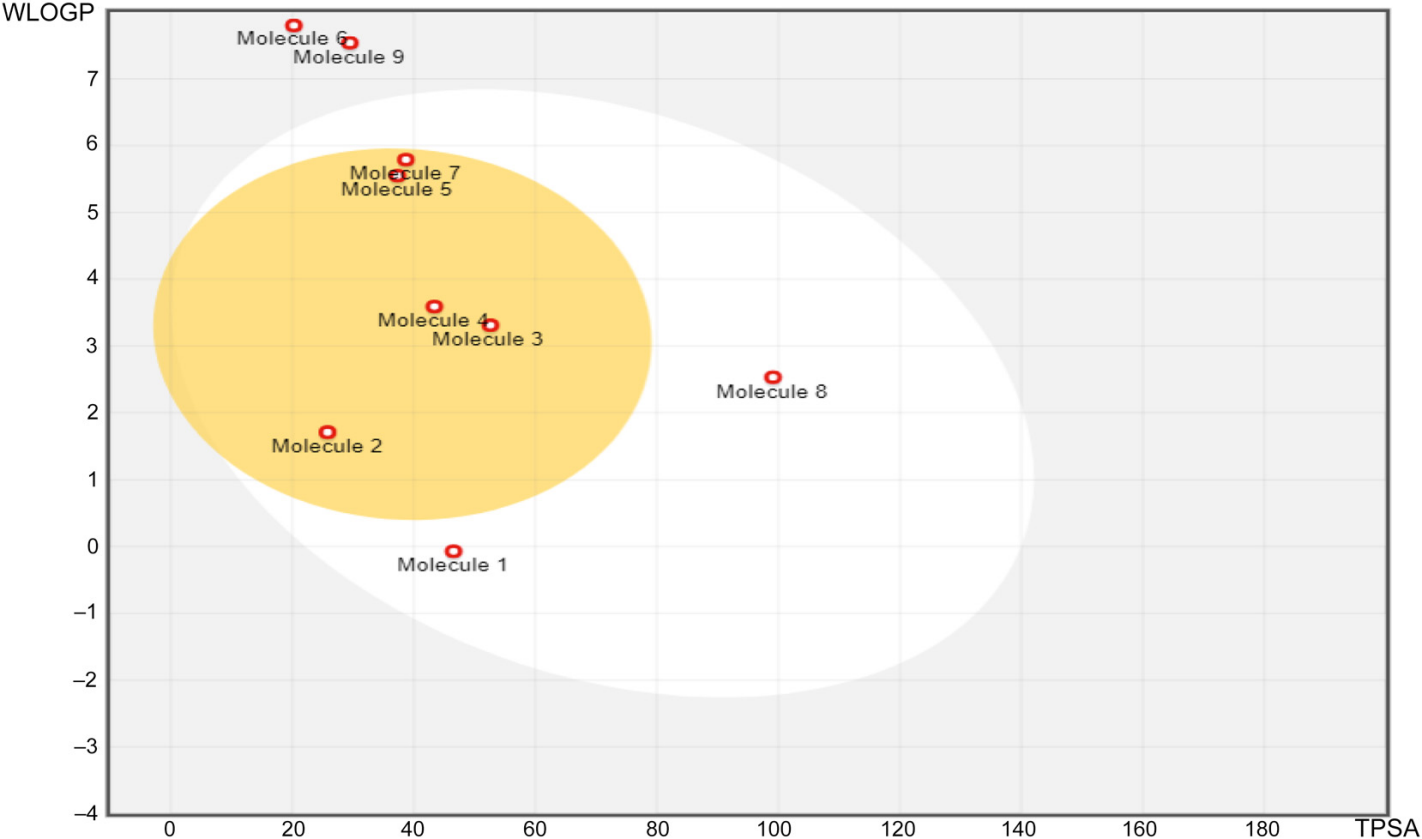
BOILED-Egg representation. It shows passive gastrointestinal absorption (HIA) and brain penetration (BBB) with molecules in the WLOGP-versus-TPSA. Molecule 1; FDHD, molecule 2;TMP, molecule 3; phthalic acid, molecule 4; DTBO, molecule 5; palmitic acid, molecule 6; stigmasterol, molecule 7; sarsasapogenin, molecule 8; butyl citrate, molecule 9; DMDP.

The BOILED-Egg (Figure 3) model is a spontaneous and efficient, approach to predict the passive gastrointestinal absorption, which is beneficial for drug discovery and development. The white region represents the area where molecules are more likely to be absorbed by the gastrointestinal tract due to their greater extent of absorption. On the other hand, the yellow region (yolk) indicates the space with the highest possibility of permeating to the brain. Cytochrome p450 (CYP) isoenzymes metabolise a significant portion (50–90 %) of medicinal compounds, utilising its five primary isoforms (CYP3A4, CYP2C9, CYP2D6, CYP2C19). P-gp is widely distributed throughout the intestinal epithelium, where it actively transports xenobiotics from the intestinal lumen back into the bloodstream. Similarly, P-gp is also present in the capillary endothelial cells of the brain, where it pumps xenobiotics from the brain back into the capillaries (Table 6) [21].

**Table 6:** Pharmacokinetics of ligands.

Ligands	Pharmacokinetics								
	GI absorption	BBB permeant	Pgp substrate	CYP1A2 inhibitor	CYP2C19 inhibitor	CYP2C9 inhibitor	CYP2D6 inhibitor	CYP3A4 inhibitor	Skin permeation log Kp (cm/s)
FDHD	↑	X	X	X	X	X	X	X	−6.77
TMP	↑	✓	X	X	X	X	X	X	−6.22
Phthalic acid	↑	✓	X	✓	✓	X	X	X	−5.08
DTBO	↑	✓	X	X	✓	✓	X	X	−5.28
Palmitic acid	↑	✓	X	✓	X	✓	X	X	−2.77
Stigmasterol	↓	X	X	X	X	✓	X	X	−2.74
Sarsasapogenin	↑	✓	X	X	X	X	X	X	−4.23
Butyl citrate	↑	X	X	X	X	X	X	X	−6.57
DMDP	↓	X	X	X	X	X	X	X	−3

↑: high, ↓: low, X: no, ✓: tick.

Drug likeness evaluates the probability of a molecule to be administered orally as a drug, taking into account its bioavailability. Swiss ADME applies a process of screening chemical libraries to eliminate molecules that have characteristics that are not compatible with a desirable pharmacokinetics profile (Table 7). This is done using five distinct rule-based filters developed by major pharmaceutical companies, with the aim of enhancing the quality of their own chemical collections.The Lipinski filter, developed by Pfizer, is a pioneering set of criteria known as the rule of five [24]. The Ghose approach developed by Amgen, the Veber method developed by GSK, the Egan method developed by Pharmacia, and the Muegge method developed by Bayer [20].

**Table 7:** Druglikeness of ligands.

Ligands	Number of violations					Bioavailability score
	Lipinski	Ghose	Veber	Egan	Muegge	
FDHD	0	3	0	0	1	0.55
TMP	0	1	0	0	1	0.55
Phthalic acid	0	0	0	0	0	0.55
DTBO	0	0	0	0	0	0.55
Palmitic acid	1	0	1	0	1	0.85
Stigmasterol	1	3	0	1	2	0.55
Sarsasapogenin	1	2	0	0	1	0.55
Butyl citrate	0	0	1	0	1	0.55
DMDP	1	2	0	1	1	0.55

After conducting an ADME study, a refinement process led to the exclusion of four out of the initial nine ligands due to demonstrated cytochrome (CYP) inhibition. Specifically, phthalic acid exhibited inhibitory activity on CYP1A2 and CYP2C19. Similarly, the compound DTBO demonstrated inhibitory effects on CYP2C19 and CYP2C9. Palmitic acid displayed inhibitory effects on both CYP1A2 and CYP2C9, while Stigmasterol exhibited inhibition specifically against CYP2C9.

Furthermore, DMDP was excluded from further consideration due to violations in four out of five druglikeness filters, namely Lipinski, Ghose, Egan, and Muegge. These filters are essential criteria in evaluating the drug-like properties of a compound.

With the refined ligand pool now consisting of four candidates – FDHD, TMP, sarsasapogenin, and butyl citrate – the next step involves evaluating their toxicity. This critical assessment aims to ensure that these remaining ligands not only exhibit desirable drug-like properties but are also devoid of potential harmful effects that could hinder their suitability for further development. The toxicity evaluation will contribute valuable information to guide subsequent decisions in the drug development process.

#### 3.2.3 Toxicity prediction

The anticipation and assessment of potential harmful effects of compounds are integral components in the meticulous design and development of drugs. To address this critical aspect, the ProTox-II tool (https://tox-new.charite.de/), is employed for the purpose of conducting thorough toxicity analysis.

In the toxicity assessment of the four remaining ligands, it was revealed that three exhibited toxic effects. However, FDHD emerged as a non-toxic compound (Table 8). Consequently, given its favorable safety profile, the non-toxic ligand was deemed suitable for further in-depth analysis.

**Table 8:** Toxicity of ligands.

Ligands	Hepatotoxicity	Carcinogenicity	Immunotoxicity	Mutagenicity	Cytotoxicity
FDHD	X	X	X	X	X
TMP	X	✓	X	X	X
Sarsasapogenin	X	X	✓	X	X
Butyl citrate	X	✓	X	X	X

X: inactive, ✓: active.

### 3.3 Standard drug

#### 3.3.1 Drug structure retrieval

The freely accessible chemical database, ChemSpider (http://www.chemspider.com/), has been employed to retrieve the three-dimensional structure of Clomiphene citrate (Table 9).

**Table 9:** Standard drug structure.

Standard drug	Molecular formula	Molecular weight	Drug structure
Clomiphene citrate	C_32_H_36_ClNO_8_	598.083 Da	**Figure:** 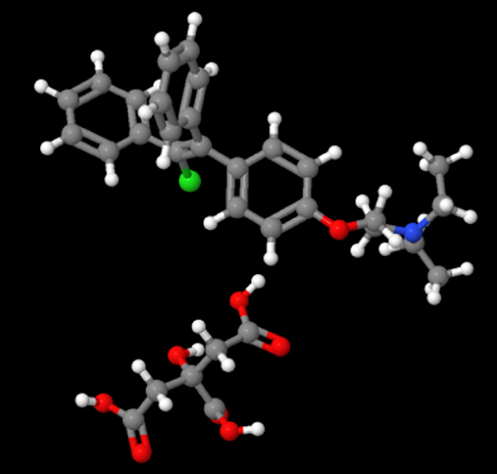

#### 3.3.2 ADMET properties

SwissADME (http://www.swissadme.ch/) is utilised for the analysis of the Absorption, Distribution, Metabolism, and Excretion (ADME) properties of Clomiphene citrate. SwissADME stands as a freely accessible tool that plays a pivotal role in providing comprehensive information across various domains crucial to medicinal chemistry. The tool offers insights into physicochemical factors (Table 10), lipophilicity (Table 11), water-solubility (Table 12), pharmacokinetics (Table 13), drug-likeness (Table 14), and other pertinent parameters [25]. By leveraging SwissADME, researchers gain valuable data that aids in the evaluation and optimization of potential drug candidates during the early stages of drug development.

**Table 10:** Physicochemical properties of clomiphene citrate.

Physicochemical properties	Results
Formula	C_32_H_36_ClNO_8_
Molecular weight	598.08 g/mol
Number of heavy atoms	42
Number of aromatic heavy atoms	18
Number of rotatable bonds	0.28
Fraction Csp3	14
Number of hydrogen bond acceptor	9
Number of hydrogen bond donor	4
Molar refractivity	161.99
Topological polar surface area	144.60 Å^2^

**Table 11:** Lipophilicity of clomiphene citrate.

Lipophilicity	Results
Log Po/w (iLOGP)	4.53
Log Po/w (XLOGP3)	2.95
Log Po/w (WLOGP)	5.31
Log Po/w (MLOGP)	2.93
Log Po/w (SILICOS-IT)	6.50
Consensus Log Po/w	4.45

**Table 12:** Water solubility of clomiphene citrate.

Water solubility	Results
Log S (ESOL)	−4.80
Solubility	9.48e−03 mg mL^−1^; 1.59e−05 mol L^−1^
Class	Moderate solubility
Log S (Ali)	−5.65
Solubility	1.34e−03 mg mL^−1^; 2.24e−06 mol L^−1^
Class	Moderate solubility
Log S (SILICOS-IT)	−9.52
Solubility	1.79e−07 mg mL^−1^; 2.99e−10 mol L^−1^
Class	Poorly solubility

**Table 13:** Pharmacokinetics of clomiphene citrate.

Pharmacokinetics	Results
GI absorption	↓
BBB permeant	X
P-gp substrate	X
CYP1A2 inhibitor	X
CYP2C19 inhibitor	X
CYP2C9 inhibitor	X
CYP2D6 inhibitor	✓
CYP3A4 inhibitor	X
Log Kp (skin permeation)	−7.85 cm/s

↓: low, X: no, ✓: tick.

**Table 14:** Druglikeness of clomiphene citrate.

Druglikeness	Results
Lipinski	Yes; 1 violation: MW > 500
Ghose	No; 3 violations: MW > 480, MR > 130, #atoms > 70
Veber	No; 2 violations: Rotors > 10, TPSA > 140
Egan	No; 1 violation: TPSA > 131.6
Muegge	Yes
Bioavailability score	0.56

**Figure 4:**
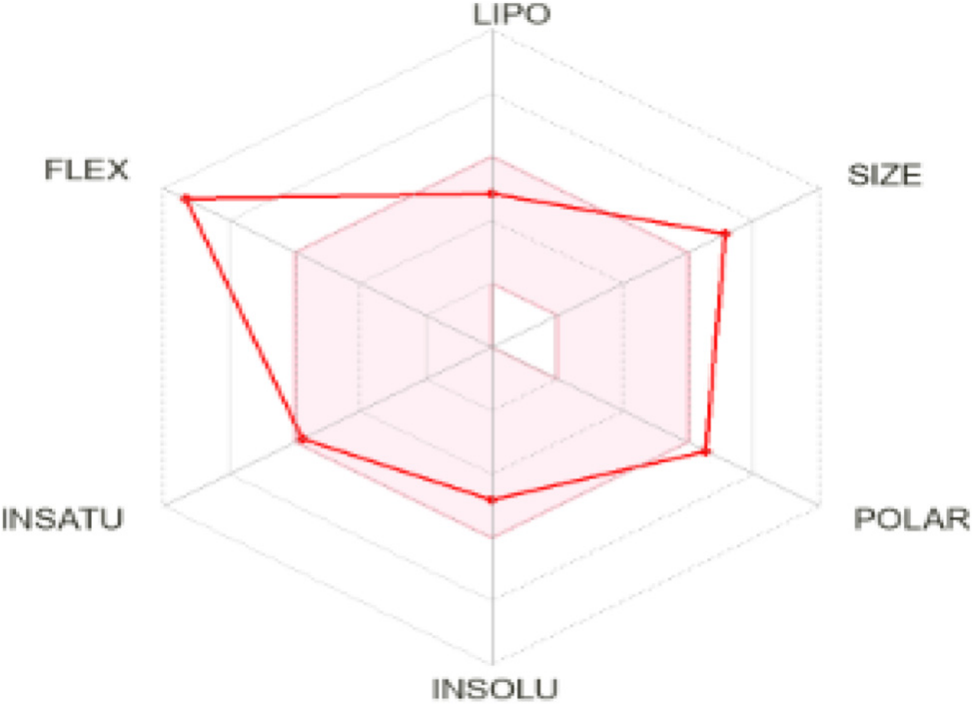
Bioavailability radar of clomiphene citrate.

**Figure 5:**
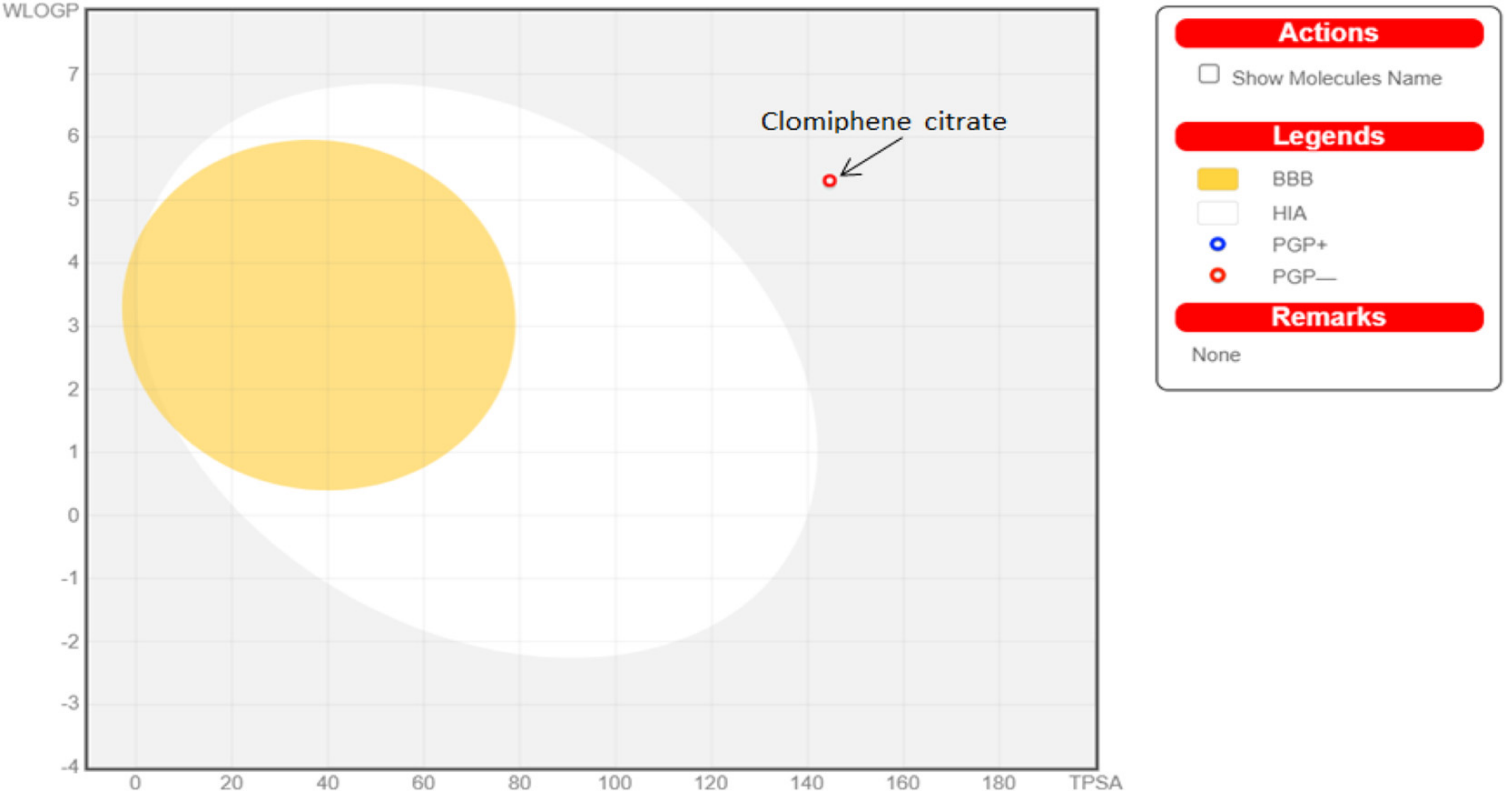
Boiled egg representation of clomiphene citrate.

Moreover, SwissADME goes beyond providing static information; it offers free access to a set of efficient predictive models, including the Bioavailability Radar (Figure 4), iLOGP, and BOILED-Egg (Figure 5) [20]. These predictive models enhance the tool’s utility by allowing researchers to anticipate crucial aspects such as bioavailability and lipophilicity, contributing to more informed decision-making in the drug development process.

In conjunction with SwissADME, the ProTox-II tool (https://tox-new.charite.de/) is employed to perform toxicity analysis specifically for the standard drug Clomiphene citrate, as outlined in Table 15. This dual-tool approach enables a comprehensive evaluation of both the physicochemical properties and potential toxic effects associated with Clomiphene citrate, providing a well-rounded understanding essential for advancing its development and application in medicinal contexts.

**Table 15:** Toxicity of clomiphene citrate.

Toxicity	Prediction
Hepatotoxicity	X
Carcinogenicity	X
Immunotoxicity	✓
Mutagenicity	X
Cytotoxicity	X

X: inactive, ✓: active.

### 3.4 Active site determination

**Figure 6:**
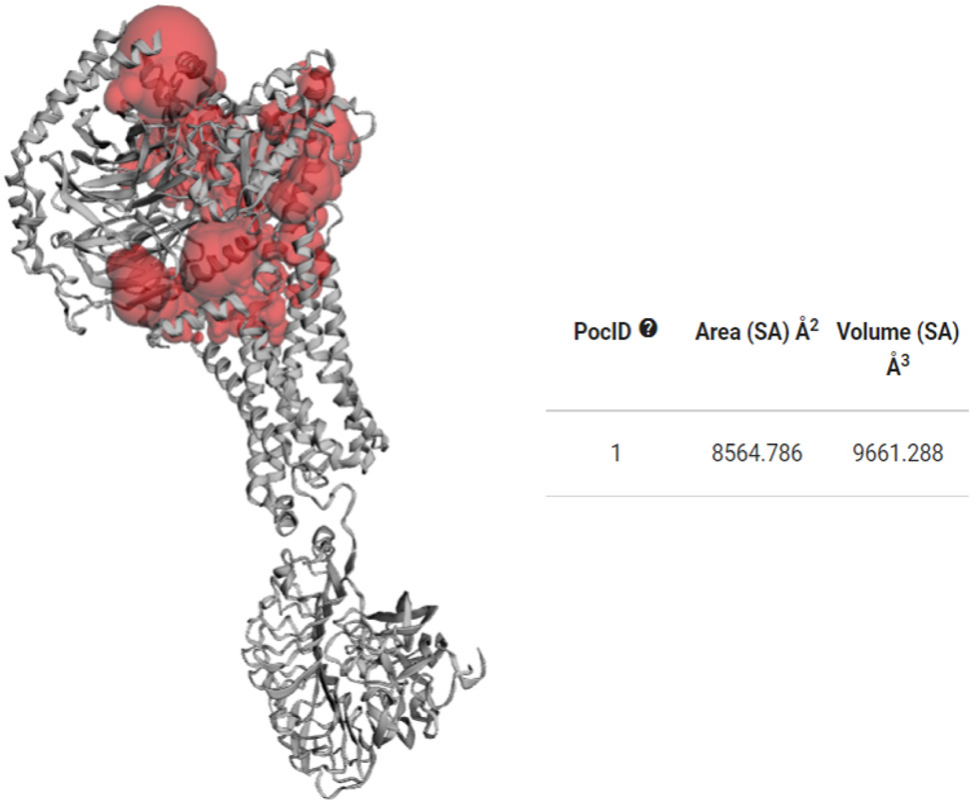
Active site of LHR.

**Figure 7:**
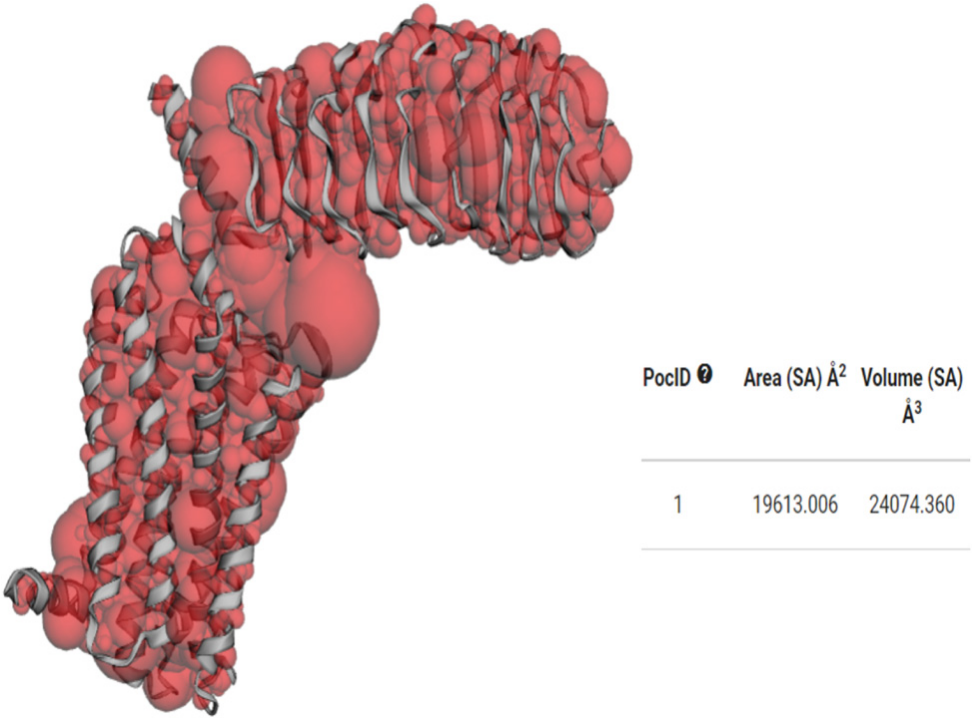
Active site of FSHR.

The active sites of the target proteins LHR (Figure 6) and FSHR (Figure 7) have been identified with Castp (http://sts.bioe.uic.edu/).

### 3.5 Molecular docking

Molecular docking is a computational approach used to determine the structure of compounds formed by two or more different molecules. The goal of docking investigations is to predict the intended three-dimensional structures. The act of docking only produces appropriate incentive structures. The scoring algorithms are utilized to sort these possibilities and identify the structures that are most probable to exist in nature [26].

The ligands and drug are subjected to docking with the target proteins using CB-Dock (http://cao.labshare.cn/cb-dock/), and the resulting protein interactions are visualized using BIOVIA Discovery Studio Visualizer [19].

#### 3.5.1 Target proteins and ligand

The selected target proteins are LHR and FSHR that are docked with FDHD.

**Figure 8:**
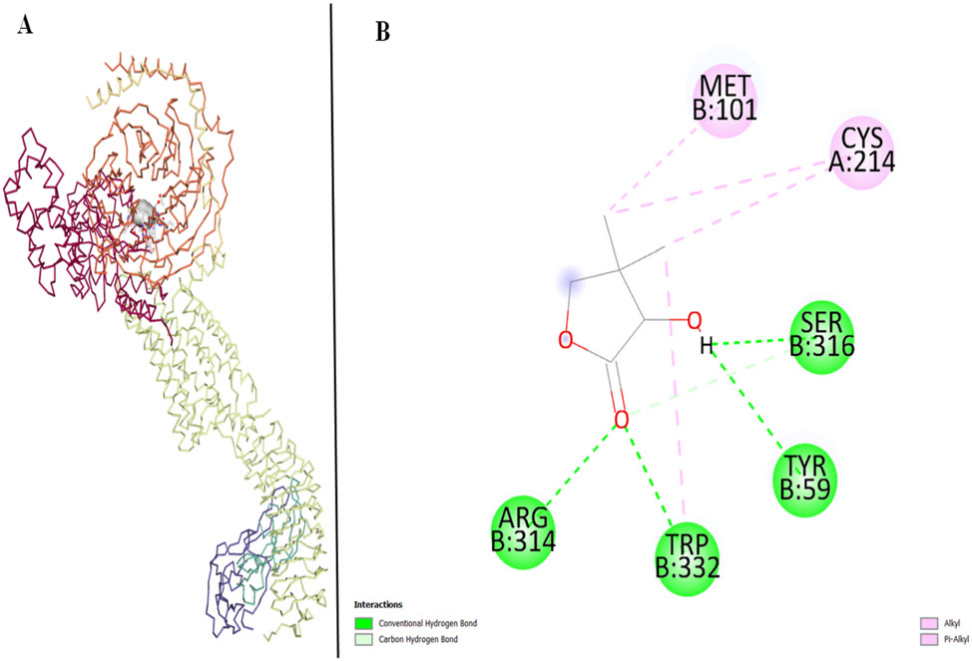
LHR target protein and ligand. (A) Molecular docking of LHR and ligand. (B) Protein interactions of LHR and ligand.

LHR has the vina score of −5 and the ligand interacts with 6 amino acids of LHR target protein. Among these interactions, Methionine (MET) and Cysteine (CYS) contribute to Alkyl and Pi-Alkyl interactions, suggesting involvement in hydrophobic contacts and interactions with aromatic rings. Tryptophan (TRP), Tyrosine (TYR), and Arginine (ARG) are associated with Conventional Hydrogen Bonding, wherein hydrogen atoms are shared between the ligand and these amino acid side chains. TRP also represents Alkyl/Pi-Alkyl interaction. Serine (SER) exhibits a multifaceted interaction, participating in both Conventional Hydrogen Bonding and Carbon Hydrogen Bonding with the ligand. This dual interaction involves hydrogen bonds, with the additional presence of carbon atoms, enhancing the complexity of the ligand-protein binding (Figure 8).

**Figure 9:**
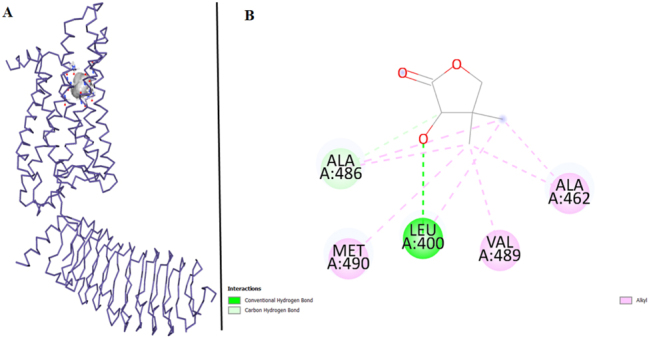
FSHR target protein and ligand. (A) Molecular docking of FSHR and ligand. (B) Protein interactions of FSHR and ligand.

On the other hand, FSHR has the vina score of −4.4 and ligand interacts with 5 amino acids of FSHR. Leucine (LEU) is involved in Conventional Hydrogen Bonding, highlighting its role in forming hydrogen bonds with the ligand. Carbon Hydrogen Bonding is observed specifically with Alanine (ALA) at position A:486, indicating a unique interaction pattern involving carbon atoms. Furthermore, both ALA A:486 and A:463 contribute to Alkyl interaction, suggesting their participation in hydrophobic contacts. Additionally, Methionine (MET) and Valine (VAL) exhibit Alkyl interaction, signifying their involvement in the ligand-protein complex through hydrophobic interactions (Figure 9).

#### 3.5.2 Target proteins and drug interaction

The target proteins LHR and FSHR are docked with standard drug clomiphene citrate, used for the management of PCOS.

**Figure 10:**
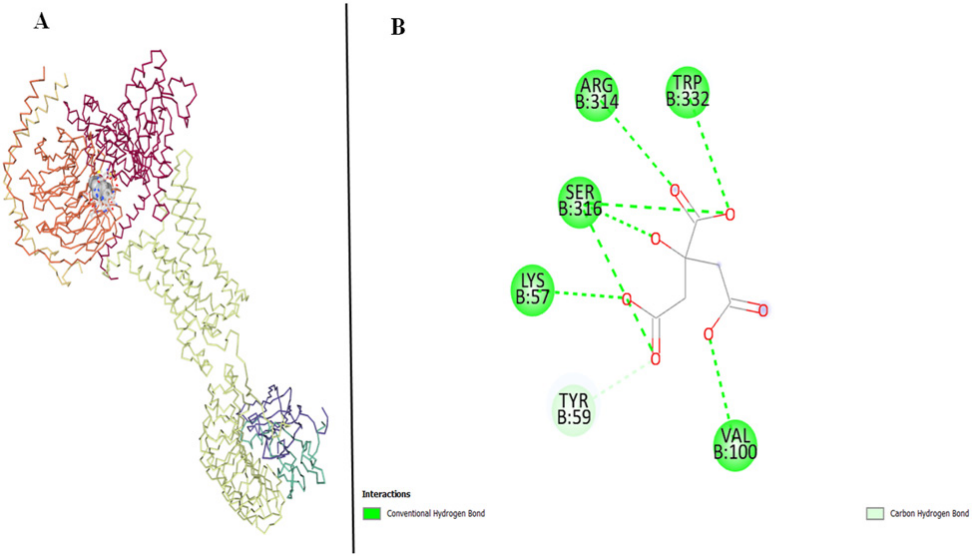
LHR target protein and clomiphene citrate. (A) Molecular docking of LHR and clomiphene citrate. (B) Protein interactions of LHR and clomiphene citrate.

LHR has the vina score of −5.9 and the ligand interacts with 6 amino acids of LHR target protein Valine (VAL), Lysine (LYS), Serine (SER), Arginine (ARG), and Tryptophan (TRP) all exhibit Conventional Hydrogen Bonding in their interactions with the ligand, indicating the formation of hydrogen bonds between the ligand and these amino acid side chains. Additionally, Carbon Hydrogen Bonding is observed specifically with Tyrosine (TYP) (Figure 10).

**Figure 11:**
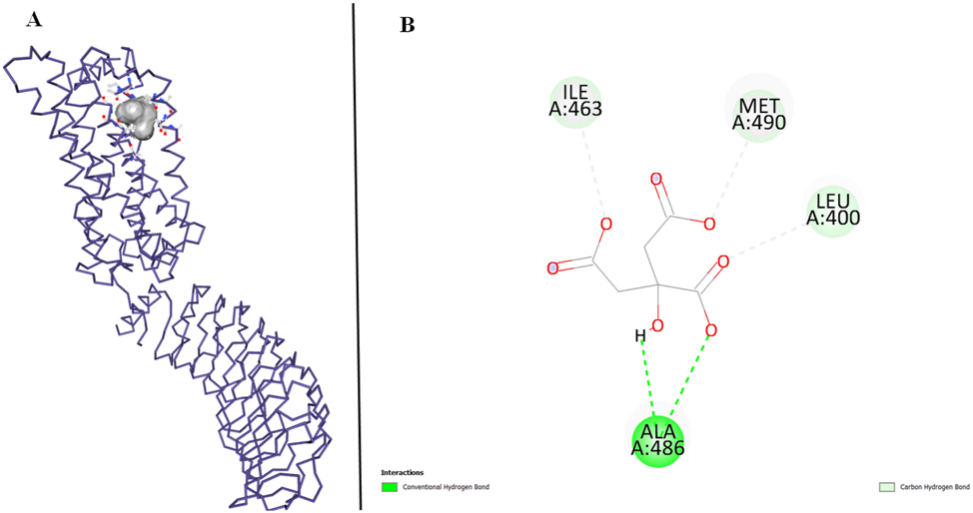
FSHR target protein and clomiphene citrate. (A) Molecular docking of FSHR and clomiphene citrate. (B) Protein interactions of FSHR and clomiphene citrate.

In contrast, FSHR has the vina score of −4.7 and ligand interacts with 4 amino acids of FSHR. Alanine (ALA) participates in Conventional Hydrogen Bonding, forming hydrogen bonds with the ligand. On the other hand, Isoleucine (ILE), Methionine (MET), and Leucine (LEU) are involved in Carbon Hydrogen Bonding. This interaction type entails hydrogen bonding with the ligand, with the additional presence of carbon atoms, contributing to the overall stability and specificity of the ligand-protein complex (Figure 11).

### 3.6 Lead compound

The ligand FDHD has emerged as the lead compound in this study. This compound not only demonstrates favorable pharmacokinetic characteristics but also exhibits a lack of toxicity. Moreover, through molecular docking, it has been established that the ligand strongly binds to the active sites of both the LHR and FSHR target proteins.

The lead compound’s favorable pharmacokinetic profile positions it as a promising candidate for further development, suggesting it may possess attributes conducive to effective absorption, distribution, metabolism, and excretion in the body. Additionally, the absence of toxicity enhances its suitability for potential therapeutic applications.

The robust binding observed through molecular docking with the active sites of LHR and FSHR further underscores the ligand’s potential as a significant player in the modulation of these target proteins.

In summary, the ligand FDHD stands out as a lead compound with a favorable pharmacokinetic profile, lack of toxicity, and strong binding affinity to the active sites of LHR and FSHR, marking it as a noteworthy candidate for further exploration in drug development and related research endeavors.

## 4 Discussion

PCOS is a prevalent reproductive endocrine disorder that impacts women in their reproductive years [1]. This enduring and heterogeneous condition manifests as irregular menstrual cycles, infertility, excessive hair growth, skin issues, and increased body weight [27]. Despite numerous epidemiological studies exploring PCOS prevalence, there is variability in their findings, influenced by factors such as race, ethnicity, study populations, and phenotypic variations [2]. A comprehensive examination of women adhering to diagnostic criteria established by the National Institutes of Health (NIH) estimates that 4–10 % of women in their reproductive age globally are affected by PCOS [3]. In Pakistan, PCOS stands out as the predominant endocrine disorder among females, with a prevalence rate of 52 % [28].

The involvement of LH and FSH from the pituitary is crucial in the pathogenesis of PCOS. In PCOS patients, there is an alteration in the LH/FSH ratio, with higher secretion of LH relative to FSH. This imbalance can lead to an increased production of androgens by theca cells and result in anovulatory cycles. The excess androgen, in turn, disrupts the regulation of female hormones, causing elevated estrogen levels, menstrual irregularities, and infertility [4]. Furthermore, LHR are predominantly found on theca cells in the ovary or Leydig cells in the testes, influencing steroid hormone biosynthesis. On the other hand, FSHR on granulosa cells in the ovary or Sertoli cells in the testes regulate the recruitment and maturation of gonadal stem cells, as well as the processing and transport of steroid hormones [29].

Presently, standard drugs utilized for treating PCOS come with specific side effects. To avoid these side effects, herbal chemicals or phytoestrogens are considered as viable alternatives for PCOS treatment [4]. *A.africanus*, a plant acknowledged in Ethiopia for its traditional use and reputed antifertility properties, belongs to the taxonomic category of Family *Liliaceae* [8]. Thus, can be a potential treatment for PCOS, offering an alternative approach with potentially fewer adverse effects.

An Insilico evaluation performed to explore the potential therapeutic effects of phytoconstituents derived from *A.africanus* against PCOS. To achieve this objective, a review of the literature led to the identification of 9 phytoconstituents associated with *A.africanus*. Subsequently, the structures of these ligands were retrieved for a comprehensive screening process. The screening involved an evaluation of the ligands ADME properties, as well as an assessment of their toxicity. The ligand exhibiting optimal pharmacokinetic characteristics and lacking toxicity, namely 2(3H)-furanose, dihydro-3-hydroxy-4,4-dimethyl, was chosen for further docking studies. In parallel, the standard drug Clomiphene citrate underwent scrutiny for its ADMET properties, revealing its role as a CYP2D6 inhibitor and its potential to induce immunotoxicity. After this, ligand and Clomiphene citrate were docked with the active sites of LH and FSH target proteins and there protein-ligand interaction was visualized. Through this analysis, the 2(3H)-furanose, dihydro-3-hydroxy-4,4-dimethyl emerged as the most potent lead compound among the tested compounds.

In comparison to related studies, *A.racemosus*, also known as Shatavari, has demonstrated effectiveness in reducing PCOS symptoms and improving follicular growth, development, and ovulation in clinical subjects [30]. Additionally, the extract from the roots of *A.officinalis* has shown positive effects on endocrine hormones, histomorphometric features of the ovary and promoting follicular development [31]. Given the promising outcomes of these studies, there is potential for *A.africanus*, belonging to a similar botanical family, to exhibit effectiveness against PCOS in clinical trials. The computational approach used here suggests that *A.africanus* may represent a potential management strategy for PCOS. Further investigation in clinical settings is warranted to validate these findings.

## 5 Conclusion

In conclusion, this study not only advances our understanding of *A. africanus’s* therapeutic potential but also lays the groundwork for subsequent research endeavors aimed at translating these discoveries into tangible clinical benefits. As we navigate the complexities of PCOS management, the insights gleaned from this study contribute to the ongoing dialogue surrounding holistic and effective treatment strategies. This study offers a promising foundation for future research on the therapeutic potential of *A. africanus* against PCOS. Despite its insightful findings, further investigations are warranted. Expanding molecular docking analyses to include additional relevant proteins and receptors, along with *in vivo* validation through clinical trials, can bridge computational predictions and real-world outcomes. Optimizing the lead compound, FDHD, and exploring combinatory approaches with existing PCOS treatments are essential. Research initiatives may delve into underlying molecular mechanisms, personalized medicine strategies, and interdisciplinary collaborations, influencing broader healthcare practices and policies. Ethical considerations also warrant careful attention, ensuring the responsible advancement of research in the pursuit of effective and ethical PCOS management strategies.

## Abbreviations

DMDP: 3-dehydro-des-N-26-methyl-dihydro-pseudotomatidine
DTBO: 7,9-die-tert-butyl-1-oxaspiro(4,5)deca-6,9-diene-2,8-dione
FDHD: 2(3H)-furanose, dihydro-3-hydroxy-4,4-dimethyl
FSH: follicle-stimulating hormone
FSHR: follicle-stimulating hormone receptor
LH: luteinizing hormone
LHR: luteinizing hormone receptor
Palmitic acid: *n*-hexadecanoic acid
PCOS: polycystic ovarian syndrome
Phthalic acid: 1,2-benzenedicarboxylic acid
TMP: tetramethylpyrazine

## References

[1] Jalilian A, Kiani F, Sayehmiri F, Sayehmiri K, Khodaee Z, Akbari M (2015). Prevalence of polycystic ovary syndrome and its associated complications in Iranian women: a meta-analysis. Iran J Reproductive Med.

[2] Bozdag G, Mumusoglu S, Zengin D, Karabulut E, Yildiz BO (2016). The prevalence and phenotypic features of polycystic ovary syndrome: a systematic review and meta-analysis. Hum Reprod.

[3] El Hayek S, Bitar L, Hamdar LH, Mirza FG, Daoud G (2016). Poly cystic ovarian syndrome: an updated overview. Front Physiol.

[4] Kamble A, Dhamane S, Kulkarni A, Potnis V (2020). Review on effects of herbal extract for the treatment of polycystic ovarian syndrome (PCOS). Plant Arch.

[5] Yang J, Chen C (2024). Hormonal changes in PCOS. J Endocrinol.

[6] Ashraf S, Nabi M, Rasool SA, Rashid F, Amin S (2019). Hyperandrogenism in polycystic ovarian syndrome and role of CYP gene variants: a review. Egypt J Med Hum Genet.

[7] Kumariya S, Ubba V, Jha RK, Gayen JR (2021). Autophagy in ovary and polycystic ovary syndrome: role, dispute and future perspective. Autophagy.

[8] Tafesse G, Mekonnen Y, Makonnen E (2006). Antifertility effect of aqueous and ethanol extracts of the leaves and roots of Asparagus africanus in rats. Afr Health Sci.

[9] Pachiappan S, Matheswaran S, Saravanan PP, Muthusamy G (2017). Medicinal plants for polycystic ovary syndrome: a review of phytomedicine research. Int J Herb Med.

[10] Amudha M, Rani S (2016). In silico molecular docking studies on the phytoconstituents of cadaba fruticosa (L.) druce for its fertility activity. Asian J Pharmaceut Clin Res.

[11] Kamel HH (2013). Role of phyto-oestrogens in ovulation induction in women with polycystic ovarian syndrome. Eur J Obstet Gynecol Reprod Biol.

[12] Kamboj A, Verma D, Sharma D, Pant K, Pant B, Kumar V (2020). A molecular docking study towards finding herbal treatment against polycystic ovary syndrome (PCOS). Int J Recent Technol Eng.

[13] Kazer R, Kessel B, Yen S (1987). Circulating luteinizing hormone pulse frequency in women with polycystic ovary syndrome. J Clin Endocrinol Metabol.

[14] Yen S, Vela P, Rankin J (1970). Inappropriate secretion of follicle-stimulating hormone and luteinizing hormone in polycystic ovarian disease. J Clin Endocrinol Metabol.

[15] Recchia K, Jorge AS, Pessôa LVF, Botigelli RC, Zugaib VC, de Souza AF (2021). Actions and roles of FSH in germinative cells. Int J Mol Sci.

[16] Richards JS, Ren YA, Candelaria N, Adams JE, Rajkovic A (2018). Ovarian follicular theca cell recruitment, differentiation, and impact on fertility: 2017 update. Endocr Rev.

[17] El-Ishaq A, Alshawsh MA, Chik ZB (2019). Evaluating the oestrogenic activities of aqueous root extract of Asparagus africanus lam in female sprague-dawley rats and its phytochemical screening using gas chromatography-mass spectrometry (GC/MS). PeerJ.

[18] Hughes E, Collins J, Vandekerckhove P (2005). Clomiphene citrate for ovulation induction in women with oligo-amenorrhoea (withdrawn paper. 1996, art. no. CD000056). Cochrane Database Syst Rev.

[19] Baroroh U, Biotek M, Muscifa ZS, Destiarani W, Rohmatullah FG, Yusuf M (2023). Molecular interaction analysis and visualization of protein-ligand docking using biovia discovery studio visualizer. Indones J Comput Biol.

[20] Daina A, Michielin O, Zoete V (2017). SwissADME: a free web tool to evaluate pharmacokinetics, drug-likeness and medicinal chemistry friendliness of small molecules. Sci Rep.

[21] Ranjith D, Ravikumar C (2019). SwissADME predictions of pharmacokinetics and drug-likeness properties of small molecules present in ipomoea mauritiana Jacq. J Pharmacogn Phytochem.

[22] Delaney JS (2004). ESOL: estimating aqueous solubility directly from molecular structure. J Chem Inf Comput Sci.

[23] Ali J, Camilleri P, Brown MB, Hutt AJ, Kirton SB (2012). Revisiting the general solubility equation: in silico prediction of aqueous solubility incorporating the effect of topographical polar surface area. J Chem Inf Model.

[24] Lipinski CA, Lombardo F, Dominy BW, Feeney PJ (1997). Experimental and computational approaches to estimate solubility and permeability in drug discovery and development settings. Adv Drug Deliv Rev.

[25] Bakchi B, Krishna AD, Sreecharan E, Ganesh VBJ, Niharika M, Maharshi S (2022). An overview on applications of SwissADME web tool in the design and development of anticancer, antitubercular and antimicrobial agents: a medicinal chemist’s perspective. J Mol Struct.

[26] Raval K, Ganatra T (2022). Basics, types and applications of molecular docking: a review. IP Int J Compr Adv Pharmacol.

[27] Motlagh Asghari K, Nejadghaderi SA, Alizadeh M, Sanaie S, Sullman MJ, Kolahi A-A (2022). Burden of polycystic ovary syndrome in the Middle East and North Africa region, 1990–2019. Sci Rep.

[28] Akram M, Roohi N (2015). Endocrine correlates of polycystic ovary syndrome in Pakistani women. J Coll Phys Surg Pakistan.

[29] Schniewind HA, Sattler L-M, Haudum CW, Münzker J, Minich WB, Obermayer-Pietsch B (2021). Autoimmunity to the follicle-stimulating hormone receptor (Fshr) and luteinizing hormone receptor (lhr) in polycystic ovarian syndrome. Int J Mol Sci.

[30] Pandey AK, Gupta A, Tiwari M, Prasad S, Pandey AN, Yadav PK (2018). Impact of stress on female reproductive health disorders: possible beneficial effects of shatavari (Asparagus racemosus). Biomed Pharmacother.

[31] Al-masoudi FJ, Jawad AK (2023). Promising histological and functional effects of asparagus officinalis L. roots extract on letrozole induced polycystic ovary syndrome in female rat. J Surv Fish Sci.

